# The Bcl-2 Family in Host-Virus Interactions

**DOI:** 10.3390/v9100290

**Published:** 2017-10-06

**Authors:** Marc Kvansakul, Sofia Caria, Mark G. Hinds

**Affiliations:** 1 Department of Biochemistry and Genetics, La Trobe Institute for Molecular Science, La Trobe University, Melbourne, VIC 3086, Australia; s.caria@latrobe.edu.au; 2 Department of Chemistry and Physics, La Trobe Institute for Molecular Science, La Trobe University, Melbourne, VIC 3086, Australia

**Keywords:** Bcl-2, apoptosis, autophagy, structural biology, poxvirus, herpesvirus, asfarvirus, iridovirus, adenovirus, host-pathogen interactions

## Abstract

Members of the B cell lymphoma-2 (Bcl-2) family are pivotal arbiters of mitochondrially mediated apoptosis, a process of fundamental importance during tissue development, homeostasis, and disease. At the structural and mechanistic level, the mammalian members of the Bcl-2 family are increasingly well understood, with their interplay ultimately deciding the fate of a cell. Dysregulation of Bcl-2-mediated apoptosis underlies a plethora of diseases, and numerous viruses have acquired homologs of Bcl-2 to subvert host cell apoptosis and autophagy to prevent premature death of an infected cell. Here we review the structural biology, interactions, and mechanisms of action of virus-encoded Bcl-2 proteins, and how they impact on host-virus interactions to ultimately enable successful establishment and propagation of viral infections.

## 1. Introduction

From the observation of specific changes in cell morphology upon cellular suicide, and ending in engulfment of the cell by phagocytes, Kerr et al. in 1972 concluded that there must be a genetically programmed form of cell death responsible for cell deletion, that they called apoptosis [1]. From these early origins, it is now recognised that apoptosis is one of a spectrum of programmed cell death (PCD) pathways that includes not only apoptosis but also autophagy, necroptosis and more specialised forms of PCD such as pyroptosis, ferroptosis, anoikis, entosis, pathanatos, netosis, and cornification [2]. The genetic and molecular basis of these different pathways are still being determined.

Correct apoptosis regulation is key to homeostasis, and regulates the clearance of cells that are no longer required in development, and are damaged, dangerous, or infected [3,4]. Apoptosis is an important regulator of the immune response, and pathogens have acquired genes that both prevent the cell from initiating apoptosis during their replicative stage, and initiating apoptosis to release their progeny. Using molecular mimicry of host proteins, pathogens have evolved mechanisms to overcome host cell defences. Such host-pathogen interactions are poorly understood, but several of these pathways are regulated by the activity of proteins of the B-cell lymphoma-2 (Bcl-2) family, a group of about 20 proteins, and numerous studies have been performed to further understand and characterise its mechanism [5,6,7,8,9]. Ultimately, the caspase cascade is activated [10], enabling disassembly of the apoptotic cell followed by its phagocyte-mediated engulfment and elimination via lysosomes [11].

**Figure 1:**
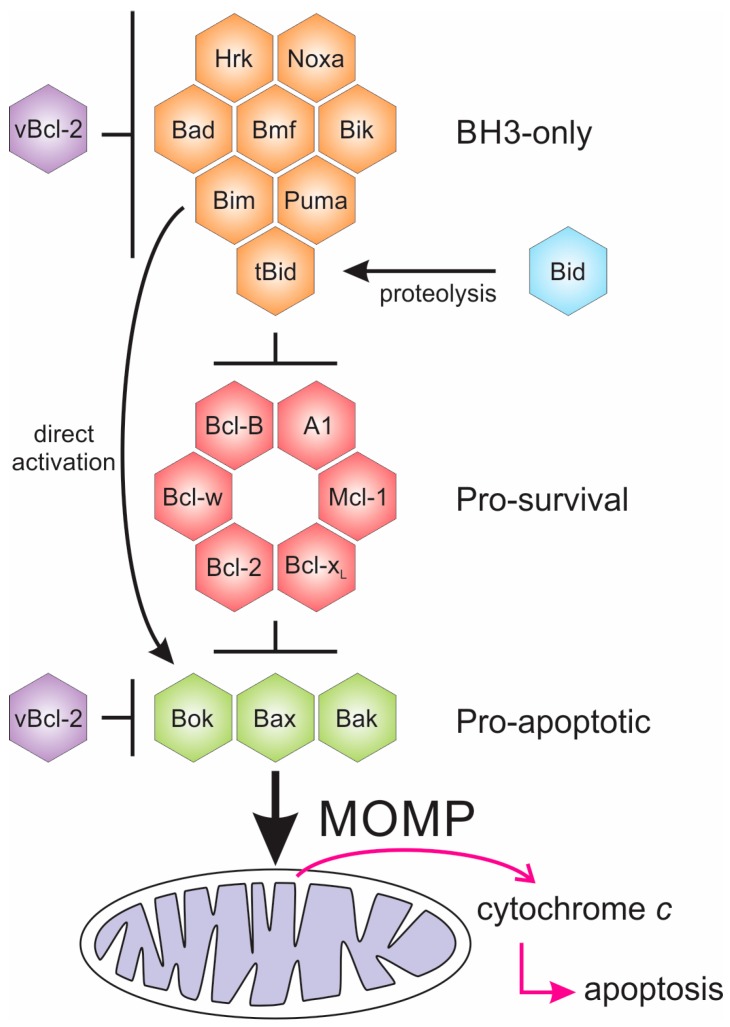
Pathways for Bcl-2 protein action. In mammals, a tripartite mechanism regulated by the Bcl-2 family controls the integrity of the mitochondrial outer membrane (MOM). Activation of Bax and Bak leads to MOM permeabilisation (MOMP), and escape of factors such as cytochrome c from the mitochondrial intermembrane space to initiate the caspase cascade that is the defining step in apoptosis. BH3-only proteins either activate Bax and/or Bak either by removal of their inhibition by pro-survival proteins, or by direct interaction. The BH3-only protein Bid is activated by proteolytic cleavage that releases its BH3-motif for interaction. Viral Bcl-2 (vBcl-2) orthologues can act on the BH3-only proteins, or directly block the action of Bax and Bak to prevent apoptosis initiation. Activation steps are shown as arrows and inhibition as bars.

Bcl-2 proteins arose early in metazoan evolution [12,13], and are characterised by the existence of short conserved sequence regions, the Bcl-2 homology (BH) motifs [14] (Figure 1). Two phylogenetically [15] and structurally [8] separate groups of proteins constitute the Bcl-2 family, one group consists of intrinsically disordered proteins (IDP) [7,16], while the other group have a globular α-helical fold structure known as the “Bcl-2 fold” [8]. The former group are IDPs and are exclusively pro-apoptotic, while the latter folded group are either pro-survival or pro-apoptotic. Both Bcl-2 family groups bear BH motifs. The pro-apoptotic BH3-only proteins bear only the BH3 motif (Figure 1), and in mammals includes the proteins Bim, Bad, Bmf, Hrk, Puma, Bik, and Noxa. The BH3-only proteins acquire secondary structure upon binding to their folded Bcl-2 targets, neutralising or activating this second group of the Bcl-2 family. The Bcl-2 fold generates a hydrophobic groove that accommodates a BH3 motif. The Bcl-2 family in mammals includes the pro-survival members Bcl-2, Bcl-x_L_, Mcl-1, Bcl-w, Bcl-B, and A1/Bfl-1 [17] while the pro-apoptotic group includes Bax and Bak, and a member Bok that as yet, has a less-well defined role [18]. The pro-apoptotic group all bear the BH3-motif, whereas the pro-survival proteins do not always bear this motif [8]. Bid bears only a BH3-motif, but has a folded α-helical structure [19,20,21], and is activated by caspase cleavage in the α1-α2 loop to form truncated Bid (tBid), the C-terminal fragment that bears the BH3-motif. It is not clear how the caspase cleaved Bid (cBid) decomposes to form N-Bid, the N-terminal fragment, and tBid, the C-terminal fragments, as cBid is stable [19]. Though tBid retains some secondary structure [22], like the other BH3-only proteins, tBid is intrinsically disordered [22,23].

**Figure 2:**
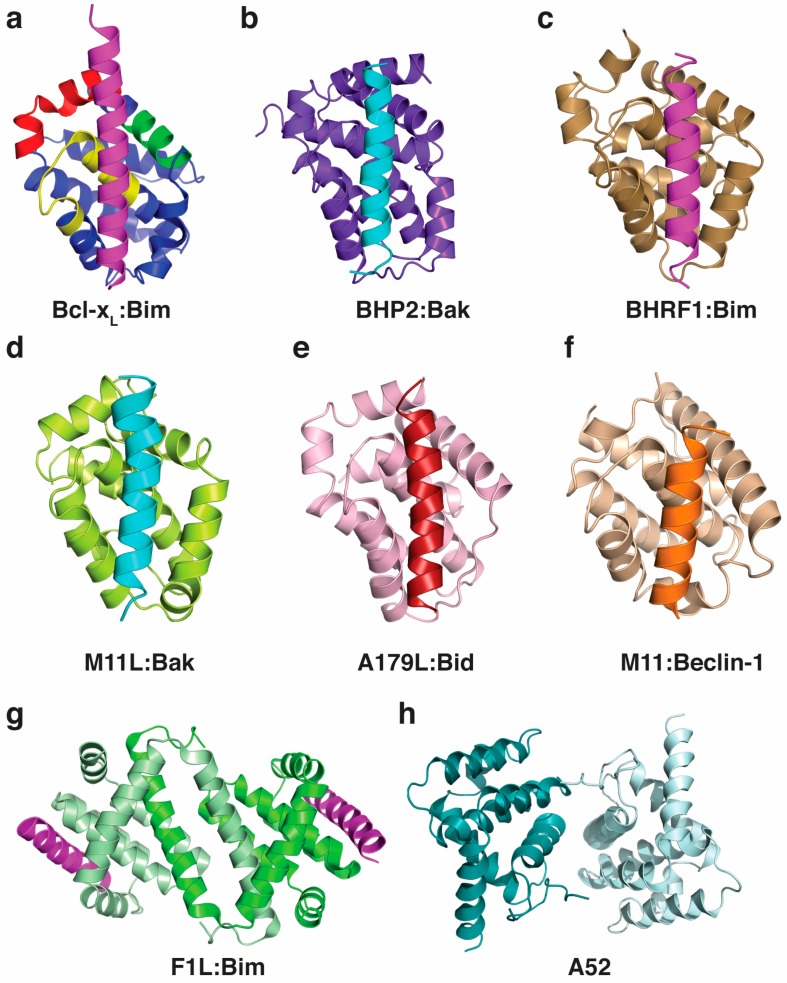
Structures of Bcl-2 family members. (**A**) Human Bcl-x_L_:Bim complex [33] (PDB ID 1PQ1); (**B**) *G. cydonium* BHP2:LB-Bak-2 complex [32] (PDB ID 5TWA); (**C**) EBV BHRF1:Bim complex [34] (PDB ID 2WH6); (**D**) Myxomavirus M11L:Bak complex [35] (PDB ID 2JBY); (**E**) African swine fever virus A179L:Bid complex [36] (PDB ID 5UA4); (**F**) Murine γ-herpesvirus 68 M11:Beclin-1 complex [37] (PDB ID 3BL2); (**G**) Vaccinia virus F1L:Bim complex [38] (PDB ID 4D2M); (**H**) Vaccinia virus A52 [39] (PDB ID 2VVW).

Bcl-2 proteins are not only pivotal in higher organisms such as mammals, worms and flies, but have been identified in evolutionary ancient species such as sponges [24,25] and hydra [26]. Key molecular and mechanistic features appear to be well preserved, with the identification of pro-survival Bcl-2 and pro-apoptotic Bak in sponges [27], along with representatives of pro-survival Bcl-2, as well as BH3-only proteins and Bak like proteins in hydra [28]. The key role of the membrane is preserved, and interactions between the pro-survival Bcl-2 and Bax-like proteins are conserved in hydra, with the Bcl-2 orthologue HyBcl-2 co-localising with HyBax on the periphery of mitochondria [29,30]. The Bcl-2 family probably forms part of a primitive immune response for cnidarians, and Bax has been shown to be upregulated in response to disease in the coral *Acropora hyacinthus* [31]. The demosponge *Geodia cydonium* Bcl-2 orthologue, BHP2, is the most ancient Bcl-2 protein to be described at a molecular level to date [32]. Structurally, the Bcl-2 fold and the BH3 motif-in-groove interaction is conserved, as shown for the sponge Bcl-2 BHP2 (Figure 2), although subtle differences allow BHP2 to discriminate between most human pro-apoptotic Bcl-2 proteins to be selective for its sponge counterparts [32].

It is now emerging that the apoptotic machinery is closely associated with other cellular regulatory pathways such as autophagy, the unfolded protein response, and endoplasmic reticulum (ER) stress signalling [40]. Viral subversion of the cellular Unfolded Protein Response (UPR) is a mechanism that is increasingly recognised as being fundamental for host immunity [41]. The UPR is a multimodal response to perturbed ER function (“ER stress”) that results from unfolded proteins accumulating in the ER faster than they are able to be folded, leading to shut down of translation, an increase in the rate of protein folding, activation of degradation pathways of the ubiquitin-proteasome or autophagy, and ultimately apoptosis if the stress is unrelieved. Thus, ER stress, autophagy, and apoptosis are all tightly linked and regulated by viruses.

The gatekeepers of mitochondrial integrity are the pro-apoptotic proteins Bax and Bak that have overlapping roles [42]. Bax and Bak are necessary for instigation of apoptosis; however, the details of their mode of action are still disputed. In mammals, after apoptotic stimuli, cytosolic Bax migrates to the mitochondrial outer membrane (MOM) to generate pores in the mitochondrial outer membrane that allows the escape of apoptogenic factors that have activated the caspase cascade (Figure 1). The apoptotic programme is not conserved in all aspects; for example, apoptotis in *Caenorhabditis elegans* differs from that in mammals. In *C. elegans*, there is a single folded Bcl-2 protein (CED-9) present in this organism that is associated with mitochondria [43], and it interacts with the caspase activator CED-4 to inhibit apoptosis. The CED-9:CED-4 interaction is antagonised by the BH3-only protein, Egl-1, to release CED-4 and activate the caspase CED-3 and the caspase cascade.

## 2. An Expanding Family of Viral Bcl-2 Orthologues has been Discovered

The importance of apoptosis and the Bcl-2 proteins in immune cell regulation and innate immunity responses has created an evolutionary pressure for viruses to acquire the genes for the pro-survival Bcl-2 proteins [44]. There are a multitude of large DNA viruses that mimic pro-survival Bcl-2 (vBcl-2) proteins, hijacking the intrinsic apoptotic pathway for their benefit; these are summarised in Table 1.

## 3. Membrane Interactions

The accumulation and oligomerisation of Bax and Bak at the intracellular membrane is the key event and the point of no return in apoptosis, and it is the least well understood at a molecular level [67]. vBcl-2 orthologues also play a role here, with many localising to intracellular membranes such as the mitochondrial membrane, ER and nuclear envelope in the host cell. The presence of a putative hydrophobic transmembrane (TM) region for many of the Bcl-2 family indicates the importance of this interaction, though the exact molecular mechanisms remain ill-defined, Bax and Bak accumulate on the mitochondrial surface and ultimately lead to its disruption and leakage. Viral control over membrane disruption is thus crucial to maintaining the host cell viability for replication [68].

The folded Bcl-2 proteins are partitioned between the cytosol and intracellular membranes and trafficking between the two environments occurs. Differences in cytosol-membrane partitioning are dependent on their rate of translocation, which is in turn dependent on their TM regions [69]. Bak and Bcl-2 are predominantly membrane-associated [70,71], while others, including Bax and Bcl-x_L_ are predominantly cytosolic but become membrane integrated after an apoptotic stimulus [72,73]. Trafficking of Bax and Bak between the membrane and cytosol is a process dependent on Bcl-x_L_ and Bcl-2 [69,74,75,76]. In addition to the requirement for the TM region the interaction between the pro-survival and proteins requires an exposed BH3-motif on Bax and Bak, a process that necessarily requires a conformational change from their solution conformation where the key residues of the BH3-motif are buried [77]. This suggests an interaction of the BH3 motif of Bax or Bak in the groove of the pro-survival protein, although this was one of the first interactions observed in the Bcl-2 family [78] there remains a deficit of structural data on this interaction.

BH3-only proteins are also associated with intracellular membranes (see [7] for a discussion), some such as Bik bear hydrophobic C-terminal regions suggestive of membrane interacting proteins. The interaction with BH3-only proteins releases the TM region from the BH3-binding groove in pro-survival proteins [79,80], potentially releasing it for membrane binding. Full biological activity of pro-survival Bcl-2 is not observed if the C-terminal region is truncated from these molecules even though binding to their BH3-targets is improved [79]. The same behaviour has been observed for viral Bcl-2 orthologues, where C-terminal deletions reduce the pro-survival activity (See Opgenorth et al. 1992 [81] for M11L truncation). Similarly, deletion of the C-terminal TM region of Bax impairs its membrane localisation and biological activity [82]. A combination of biophysical, biochemical and genetic studies have shown that there is multiple redundancy in the interactions [17,83,84]. Several models have been put forward for BH3-only protein PCD activation but most BH3-only proteins are able to activate Bax or Bak [85]. Thus, the membrane interaction is critical to the pro-survival or pro-apoptotic activity of the Bcl-2 family and further complicates an already complex multilevel-redundant regulation mechanism for mammalian apoptosis.

Many vBcl-2 orthologues, including the first viral Bcl-2 orthologue found, adenovirus E1B 19K, though without an obvious hydrophobic C-terminal region, are closely associated with intracellular membranes, the ER and nuclear envelope [86] and the association with membranes is required for its function [87]. Frog virus 3 Bcl-2 orthologue 97R localises to the ER and deletion of the C-terminal 29 residues inactivates the protein [88]. The African swine fever virus Bcl-2 orthologue A179L, is closely associated with viral factories, and though it lacks an obvious TM region, it is associated with the ER and mitochondrial membranes. However, the mutant G85A A179L loses its ability to keep cells alive, but also associates with ER membranes [89], probably indicating that a competent BH3-binding is required for ER association, as this mutant destroys binding to the BH3-only proteins [36]. EBV BHRF1 is associated with membranes [47]. Combined, these features attest to the importance of membrane association or integration to the activity of the folded Bcl-2 proteins, including those encoded by viruses.

Though many questions remain about the exact nature of the molecular assemblies of Bax and Bak that disrupt the MOM, a more consistent mechanism is now emerging where Bax and Bak undergo a series of conformational changes to form high-order aggregates to create the membrane disrupting pores [90]. However, in solution Bax is a monomeric and relatively rigid protein with little evidence of conformational mobility [77,91], a finding consistent with fluorescence cross correlation studies showing that Bax associates with mitochondria prior to oligomerisation [92] into ring-like pores [93,94]. In a defined system consisting of only cBid, Bax and Bcl-x_L_, Bleicken et al. showed that the interactions between these apoptotic regulators is spatially regulated. When embedded in the membrane, Bax is proposed to form a positive feed-back loop recruiting Bax, and Bcl-x_L_ inhibits this process by preventing Bax oligomer growth and translocating membrane Bax to the cytosol [92]. Accumulating evidence suggests that the TM regions are intermolecular interaction sites. Andreau-Fernandez et al. showed that the TM region of Bax interacts with the TM regions of Bcl-2 and Bcl-x_L_ [95]. Earlier structural studies on Bcl-w where a near-full-length sequence was well behaved [80], showed that like Bax [77] the C-terminal tail lies in the BH3-binding groove. Furthermore, the presence of the TM region in Bcl-x_L_ [79,96] and Bcl-w [80] reduces their affinity for BH3-motifs. In the case of Bcl-x_L_ is dimeric when the TM region is present [92] and monomeric in its absence, as shown by structural studies [97]. Nuclear Magnetic Resonance (NMR) investigation of the Bcl-x_L_:membrane interaction showed that it has an α-helical C-terminal tail that anchors the folded globular Bcl-2 domain head to the membrane [98]. BH3-ligand displacement of the C-terminal residues of the α9 residues from the groove of Bcl-w renders them unstructured in solution [80], and a likely mechanism to drive Bcl-w to the membrane [79]. Biochemical studies showed that like Bcl-x_L_, Bak has a transmembrane C-terminal anchor [99]. Combined, these studies indicate that the C-terminal region of the Bcl-2 fold are not by-standers, and play an important role in not only membrane targeting and anchoring, but also the protein-protein interactions of these molecules. The observation that the viral pro-survival proteins mimic these membrane localisation and activities suggests the importance of modulating PCD in infected cells.

Other models for the membrane oligomerisation include an initial dimerisation of Bax or Bak [100] prior to their oligomerisation at the membrane surface by unfolding an interaction. In this model, it is proposed that membrane rupture is caused by disordered clustering of Bak or Bax dimers. A “hit and run” mechanism has been suggested, where an initial weak interaction induces subsequent conformational changes in Bax or Bak. A second site has been proposed for binding BH3-only proteins on Bax, though it is a low affinity interaction that initiates apoptosis [101]. Structural investigation of detergent treated Bax in the presence of BH3-motifs gave a symmetrical Bax dimer with the BH3 bound in the groove; this was proposed to be the active from of Bax that further oligomerises to form pores [102]. Structures of Bim and Bid BH3-motifs in the groove of domain swapped dimer have been determined [103], similar domain-swapped dimers have been observed for Bcl-x_L_, that also retain the ability to bind BH3 motifs [104]. A caveat on these studies is that they were performed with C-terminally truncated Bax and may not reflect membrane interactions in their entirety [92]. Further studies will be required to elucidate the exact mechanisms.

## 4. Viral Bcl-2-mediated Subversion of Programmed Cell Death

Considering the importance of Bcl-2 proteins in regulating apoptosis, as well as autophagy in higher organisms [11,84], it is unsurprising that numerous viruses have acquired sequence, functional and structural homologs of Bcl-2 to subvert host apoptosis as well as autophagy signalling for their own ends. Prevention of premature host cell death during the initial stages has been shown to be critical for successful infection of EBV [68] and demonstrates the pivotal role that disarming of host apoptotic defences plays in preventing clearance of virus to enable successful infection and propagation. However, whilst prevention of premature host cell apoptosis is highly desirable, ultimately, viruses also rely on host cell apoptosis at a later stage to aid viral dissemination, for example an avian reovirus triggers apoptosis to enable optimal release and dissemination of viral progeny [105].

The earliest identified virus encoded Bcl-2 homologs were E1B 19K from adenovirus and BHRF1 from EBV which both display substantial sequence identity (18% and 16% identical to human Bcl-2 respectively) to mammalian Bcl-2 and contain the hallmark BH1 and BH2 motifs. Functional studies of E1B 19K determined that it is a potent inhibitor of apoptosis that is induced by stimuli including Fas ligand, TNFα, and adenoviral infection. Mechanistically, E1B 19K engages Bax [106], Bak [107] and Bik [108], and is functionally interchangeable with Bcl-2 during adenovirus infection and transformation [46].

One mechanism for modulating apoptosis is to manipulate the BH3-only and Bax family through interaction in the binding groove (Figure 2). This interaction has now been extensively studied and binding affinities measured (Table 2), but the implications of binding specific BH3-motif bearing proteins is not always clear. For example, Bim is a universal Bcl-2 binder and binds all mammalian pro-survival proteins with relatively high affinity, yet is not bound by all vBcl-2 proteins (for example the variola virus F1L). Some viral Bcl-2 proteins are capable of binding nearly all pro-apoptotic proteins (e.g., A179L, FPV039), while others have a much more specific ligand range (Table 2). The specificities of the viral Bcl-2 proteins for their BH3-targets have been determined (Table 2), and in general, these interactions are of lower affinity than the pro-survival Bcl-2, probably reflecting a balancing act by the virus, as they need to block apoptosis during their replicative stage; however apoptosis is necessary for the escape of viral progeny on maturation. The cell type specificity of the virus also probably plays a role in deciding which host Bcl-2 proteins are inhibited, and this is an area for further investigation.

## 5. *Herpesviridae*-Encoded Bcl-2 Homologs

Many members of the *herpesviridae* encode Bcl-2 like proteins. Epstein-Barr virus (or human herpesvirus 4) is a large DNA virus belonging to the *γ-herpesviridae* and harbours two Bcl-2 homologs, BHRF1 and BALF1. BHRF1 was shown to be an enhancer of cell survival [47]. Biochemical and structural studies revealed that BHRF1 adopts a Bcl-2 fold [34,109] and is bound to the BH3-only proteins Bim, Bid, and Puma, as well as Bak and Bax [34,110] (Table 2). Mechanistically, BHRF1 was shown to rely on the sequestration of Bim [111] and Bak [34] to inhibit apoptosis, and to confer chemoresistance in a Burkitt lymphoma mouse model, similar to Bcl-2 [34]. BHRF1 was also shown to be constitutively overexpressed in a sub-set of EBV transformed B-cells, thus rendering them resistant to apoptosis [112]. The function of a second EBV-encoded Bcl-2 homolog, BALF1, remains controversial. Initial data suggested that BALF1 acts as a pro-survival Bcl-2 protein [113], however a second report showed that BALF1 is pro-apoptotic and inhibits the other EBV-encoded pro-survival protein BHRF1 [114]. Subsequently, others reported that both BHRF1 and BALF1 are required for successful EBV-induced B-cell transformation [68]. The identification of BHRF1 in transformed B-cells sparked interest in developing antagonists against BHRF1 for targeted cancer therapy, and the feasibility of such an approach was recently demonstrated via the use of an engineered protein that bound BHRF1 with picomolar affinity [115]. No small molecule antagonists for BHRF1 have been reported yet; however their development is underway [116].

Kaposi’s sarcoma herpesvirus (KSHV or human herpesvirus 8) is also a large DNA virus and a member of the *γ-herpesviridae.* KSHV encodes a Bcl-2 homolog, Ks-Bcl-2 [117] that adopts a Bcl-2 fold [49] and is able to bind Bim, Bid, Bik, Bmf, Hrk, Noxa, and Puma [110] (Table 2). Conflicting data exist for binding of Bax and Bak, with one report indicating that neither bind Ks-Bcl-2 [48], whereas a subsequent study revealed a high affinity interaction for Bak (50 nM) and moderate affinity for Bax (980 nM) [109]. During viral infection, Ks-Bcl-2 appears to play a pivotal role in completion of the lytic cycle, as a Ks-Bcl-2 deletion virus of KSHV does not complete the lytic replication cycle. Interestingly, the Ks-Bcl-2-related ORF16 from rhesus rhadinovirus is able to functionally replace Ks-Bcl-2 during the lytic cycle, in contrast to other endogenous mammalian Bcl-2 proteins, such as Bcl-x_L_ or other herpesvirus-encoded vBcl-2 proteins including M11 and vMIA [118]. Another rhadinovirus, herpesvirus saimiri, also encodes a Bcl-2 homolog named ORF16 [119], which was shown to be anti-apoptotic and bound Bak and Bax in pull-down assays.

Murine γ-herpesvirus 68 encodes M11 [120] and was identified as an inhibitor of Fas and TNF induced apoptosis [45,121], but biochemical studies demonstrated binding to Bim, Bid, Bmf, Noxa, Puma, and Hrk, as well as Bak and Bax via the canonical ligand binding groove of its Bcl-2 fold [37]. Thus, M11 can inhibit the major mitochondrial pathways to apoptosis, however functional studies [37,122] indicate that the mitochondrial pathway may not be the primary target for M11 (see below).

Cytomegalovirus (CMV or human herpesvirus 5) is a large DNA virus belonging to the *β-herpesviridae.* CMV encodes proteins that directly target host pro-apoptotic proteins Bax and Bak, but appear to be neither sequence nor structural homologs of Bcl-2. Human CMV encodes vMIA, which has been shown to inhibit Bax [123] and Bak oligomerisation [124,125]. Interestingly, the interaction of vMIA with Bax does not involve the canonical Bcl-2 ligand binding groove. Unexpectedly, vMIA bound to an alternative binding site distinct from the canonical BH3 binding groove in Bax, which was mapped using NMR to define an interaction site comprising primarily of the loops connecting α1-α2, α3-α4, and α5-α6. Furthermore, the vMIA-Bax interaction was of high affinity with a K_d_ of 22 nM [126]. Whilst human CMV vMIA appears to be able to neutralise both Bax and Bak, in mouse CMV Bax and Bak are neutralised by two proteins with single specificity [127]. MCMV-encoded m38.5 has been shown to be mitochondrially localised, and to inhibit Bax activation [128,129], whereas Bak inhibition is achieved via m41.1 [130,131].

Amongst the α-herpesviruses, a virus-encoded homolog of the endogenous turkey pro-survival Bcl-2 protein NR13 [132], Bcl-B [133], Boo, Diva, or NRH [134] in mammals, has a Bcl-2 fold [135,136] and has been termed vnr-13 [50]. Though little is known about vnr-13, it was shown to localise to the outer mitochondrial membrane, and inhibit apoptosis after serum deprivation [50].

## 6. *Poxviridae*-Encoded Bcl-2 Homologs

The *poxviridae* encompass a number of families that encode for Bcl-2 proteins. Vaccinia virus is a large DNA virus, the prototypical member of the orthopoxviruses, and encodes F1L, a potent inhibitor of intrinsic apoptosis [55,56] that displays no recognisable sequence identity to Bcl-2. Structural studies revealed that F1L adopts an unusual Bcl-2 fold featuring a domain-swapped dimer configuration, in marked contrast to mammalian pro-survival Bcl-2 proteins, which are all monomeric [57,143]. Furthermore, F1L only bound a highly restricted subset of pro-apoptotic Bcl-2 including Bim [56,144] and Bak [145,146] (Table 2), and was shown to inhibit Bak activation by functionally replacing Mcl-1 during infection [147]. Although F1L is also able to inhibit Bax-mediated apoptosis [144], this activity is likely via an indirect mechanism as F1L does not engage Bax in a cellular context. Like other Bcl-2 family proteins F1L is localised to mitochondrial membranes through its C-terminal residues, and this region is necessary for full pro-survival activity [148]. Mechanistically, the interaction of F1L with Bim was identified as the primary mechanism underlying F1L-mediated inhibition of apoptosis in the context of a live viral infection [143]. Interestingly, the F1L homolog in variola virus, the causative agent of smallpox and another member of the *orthopoxviridae*, appears to utilise a different mechanism for apoptosis inhibition, despite adopting a near identical structure and sequence [58]. Unlike its vaccinia virus counterpart, variola virus F1L only binds Bid, Bak, and Bax, and not Bim (Table 2), and only inhibits Bax-mediated apoptosis [58]. A homolog of vaccinia virus F1L found in another orthopoxvirus, Ectromelia virus EMV025, was also shown to be anti-apoptotic by inhibiting Bax and Bak activation by directly engaging Bim and Bak [59].

Myxomavirus is a member of the *leporipoxviridae* and encodes for M11L, another potent inhibitor of intrinsic apoptosis lacking detectable sequence similarity with Bcl-2 [149]. M11L is able to engage several host pro-apoptotic Bcl-2 proteins including Bak [150], Bax, Bim, and Bid [35] (Table 2). Structural studies showed that M11L adopts a compact, monomeric Bcl-2 fold [35,151] where the canonical ligand binding groove is used to engage pro-apoptotic Bcl-2 proteins [35]. Interestingly, functional studies revealed that M11L primarily acts by sequestering Bak and Bax [35], in contrast to vaccinia virus F1L which acts primarily via Bim sequestration [143].

Orf virus is a parapoxvirus and encodes a readily identifiable Bcl-2 homolog, ORFV125, that potently inhibits intrinsic apoptosis [66]. Functional studies revealed that ORFV125 interacts with several BH3-only proteins including Bim, Puma, Hrk, Bik, and Noxa as well as active Bax but not Bak [152].

Among the *avipoxviridae*, both fowlpoxvirus FPV039 and canarypoxvirus CNP058 have been shown to suppress apoptosis. FPV039 inhibits apoptosis [153] after overexpression of all BH3-only proteins [153], and was shown to adopt a Bcl-2 fold and engage all major host pro-apoptotic Bcl-2 proteins [64] (Table 2). Interestingly, the closely related canarypoxvirus CNP058 also inhibits apoptosis in transfected cells, but engaged a different subset of pro-apoptotic Bcl-2 proteins, largely with weaker affinities than FPV039 [64].

Other Bcl-2 proteins-encoded by poxviruses include sheeppoxvirus SPPV14 and deerpoxvirus DPV022. SPPV14 displayed a broader spectrum of pro-apoptotic Bcl-2 interactions by binding Bim, Bid, Bmf, Hrk, Puma, as well as Bax and Bak [60] (Table 2). In contrast, DPV022 only engaged Bim, Bax and Bak [62] (Table 2). Intriguingly, DPV022 also adopted a domain-swapped Bcl-2 fold similar to those observed for vaccinia and variola virus F1L [57,58,143], suggesting that this particular topology for Bcl-2 proteins may be more widely found in nature.

## 7. *Asfarviridae* and *iridoviridae*-Encoded Bcl-2 Homologs

African swine fever virus is a large double stranded DNA virus, the only member of the *asfarviridae*, and encodes A179L [154]. A179L adopts a Bcl-2 fold [36], and displays extreme promiscuity by binding all host pro-apoptotic Bcl-2 proteins [36,155]. A179L localised to mitochondria [89] and potently inhibits apoptosis in cell culture assays [51]. Amongst the *iridoviridae*, grouper iridovirus was shown to encode GIV66, which inhibited apoptosis in a grouper kidney cell culture model [156]. However, the structural and functional basis of GIV66-mediated apoptosis inhibition has not been established.

## 8. Other Functional Roles of Viral Bcl-2 Homologs

Although the vast majority of virus-encoded Bcl-2 proteins primarily interfere with host cell intrinsic apoptosis signalling by targeting endogenous pro-apoptotic host Bcl-2 proteins, a number of studies have revealed that vBcl-2 proteins also harbour other activities. Several vBcl-2 proteins have been shown to inhibit autophagy. Murine γ-herpesvirus 68-encoded M11 utilises the canonical Bcl-2 ligand binding groove to bind the BH3 motif of Beclin-1, a key autophagy regulator [37,122]. Another member of the *herpesviridae*, KSHV, also targets Beclin-1 using Ks-Bcl-2 [157]. However, this ability to engage Beclin-1 is not limited to the *herpesviridae*, with African swine fever A179L displaying autophagy inhibitory activity in addition to anti-apoptotic activity [89]. Adenoviral E1B 19K was also shown to bind Beclin-1, and thus inhibit autophagy [158]. However, to date, no autophagy inhibitor has been identified amongst the *poxviridae*.

Another virus-encoded Bcl-2 protein with multiple functionalities is the vaccinia virus F1L. In addition to the anti-apoptotic activity mediated by sequestering Bim, F1L was also shown to inhibit inflammasome activation [159] via an unusual unstructured N-terminal extension prior to the Bcl-2 fold [38]. In addition to the ability to mediate inflammasome activation, the N-terminus of F1L was also proposed to act as a caspase-9 inhibitor [160,161], however, a subsequent study suggested that the F1L N-terminus is not involved in apoptosis inhibition [38].

F1L is not the only vaccinia virus-encoded Bcl-2 protein with dual functionality. N1L was shown to adopt a Bcl-2 fold (albeit lacking a TM anchoring region) and inhibit both intrinsic apoptosis by targeting several pro-apoptotic Bcl-2 proteins as well as modulating NF-κB signalling. Interestingly, N1L also adopts a Bcl-2 fold with dimeric topology; however, dimerisation is not achieved via a domain swap as seen in F1L and DPV022, but rather via a novel interface centering on the α1 and α6 helices [138,162]. Furthermore, the ability to manipulate both apoptosis and NF-κB signalling is mediated via two discrete sites on N1L [163].

In addition to N1L, several other NF-κB inhibitors that adopt Bcl-2 folds have now been identified in vaccinia virus. These include B14 [39] andA52 [39], as well as A46 [164,165], A49 [166] and K7 [167]. Whilst all four proteins inhibit NF-κB, they are distinguished by substantial differences in mechanism, cellular activity, and structure. Similar to N1L, B14 and A52 form dimers utilising an interface involving α1 and α6 helices, with small but significant differences in the orientation of monomeric chains with each other within the dimers amongst the three proteins [39]. Furthermore, A46 also forms dimers, however, this involves an interface formed by α4 and α6 helices of the C-terminal Bcl-2 like domain. Intriguingly, A46 harbours an additional N-terminal domain that mediates tetramerisation of A46, thus adding an additional layer of quaternary structure-based regulation [168]. In contrast, A49 and K7 are monomeric in solution. Unlike N1L, B14, A52 and K7 do not bind pro-apoptotic Bcl-2 proteins. However, K7 harbours dual functionality that is similar to N1L, and in addition to inhibition of NF-κB, it also binds to the human DEAD-box RNA helicase DDX3 to inhibit induction of the IFN-β promoter [169,170].

## 9. Concluding Remarks

Virus-encoded Bcl-2 proteins have demonstrated the remarkable adaptability of the Bcl-2 fold, and its ability to modulate signalling that involves several cell death-associated pathways via multiple mechanisms. Whilst mammalian pro-survival Bcl-2 proteins display several distinct rules of engagement for their interactions with pro-apoptotic Bcl-2, the picture is not as clear amongst the virus-encoded homologs of Bcl-2. In mammals, key interactions between pro-survival and pro-apoptotic Bcl-2 are typically characterised by high affinities, with some such as Bcl-x_L_:Bim interactions straying into picomolar affinities. The caveat with the binding studies is that they have been performed with C-terminally truncated molecules, and probably overestimate true affinities [79,80]. Furthermore, all mammalian pro-survival Bcl-2 proteins target Bim, the sole universal pro-apoptotic BH3-only protein [135] and either Noxa or Bad, but not both [139]. In contrast, key interactions between virus-encoded pro-survival proteins tend to display weaker affinities, typically with dissociation constants (K_d_) in the nanomolar range, although the dimeric poxvirus-encoded pro-survival proteins F1L [57,58] and DPV022 [62] only display low nanomolar or micromolar K_d_ values. It remains to be determined if these markedly weaker affinities are related to the different oligomeric state of the virus-encoded proteins. Furthermore, the Bad/Noxa dyad does not apply to virus-encoded pro-survival Bcl-2, with fowlpoxvirus FPV039 [64] and ASFV A179L [36] binding both Noxa and Bad. Indeed, both of these proteins bind all major pro-apoptotic Bcl-2 proteins, another feature not previously observed amongst their mammalian counterparts. Lastly, Bim is not a universal target amongst virus-encoded Bcl-2, with variola virus F1L showing no affinity for Bim, and instead displaying weak binding to Bid [58] whilst being a potent inhibitor of Bax-mediated apoptosis in cellular assays. Overall the virus-encoded Bcl-2 pro-survival proteins display weaker affinities for host pro-apoptotic proteins. This could suggest that only small perturbations of the overall balance between pro-survival and pro-apoptotic proteins in a host cell are sufficient to impede apoptosis progression; however, the functional relevance of these lower affinities remains to be clarified.

When considering mechanisms and the long-standing debate on the precise mechanism of action of cellular pro-survival Bcl-2, current models and mechanisms have not been entirely resolved [84]. BH3-only proteins antagonise the pro-survival Bcl-2 family that in turn keep Bax and Bak in check, but they can also activate Bax and Bak (Figure 1). Direct binding to Bax and Bak has been observed though the affinities are generally low, for example, Bim binds full length Bax with a K_d_ of 3.1 μM compared with K_d_ in low nanomolar ranges for the pro-survival proteins [171]. While the exact details of the membrane pore generated by Bak and Bax remain under investigation, it is clear that the interactions at the membrane are crucial to the apoptotic response in mammals, and viruses also appear to exploit this. vMIA [172] and other viral Bcl-2 proteins translocate to the MOM, and a possible role for them would be to inhibit pore formation by either preventing pore growth through sequestration or retrotranslocation of the components into the cytosol as in the case of Bcl-x_L_ [74,92]. It is becoming apparent for the best understood virus-encoded Bcl-2 proteins that, in contrast to mammalian apoptosis, multiple mechanisms of action exist, though many of these need to be clarified with quantitative structure and binding studies that are complemented by live viral infection models. Whereas myxomavirus M11L was shown to act by sequestering Bax and Bak [35], vaccinia virus F1L was shown to only require neutralisation of Bim in a viral infection setting [143]. For EBV BHRF1, a combination of neutralisation of Bim [111] and Bak [34] was required.

When considering the role of membranes in Bcl-2 activity, and the observation that it is Bak and Bax accumulation at the membrane that is critical for intrinsic apoptosis to proceed, the association of virus-encoded Bcl-2 proteins with membranes is not unexpected. Nearly all apoptosis inhibitory vBcl-2 proteins harbour transmembrane anchoring regions to direct their subcellular localisation, chiefly to the MOM. Interestingly, different vBcl-2 proteins appear to inhibit different stages of Bax activation and translocation to the outer mitochondrial membrane [173]. E1B 19K and BHRF1, are examined for their ability to block Bax activation at different steps and thereby reveal the timing of mitochondrial changes during apoptosis. BHRF1 inhibited Bax activation but not upstream of apoptotic signalling events, whereas E1B19K permitted the initial stages of Bax activation to proceed, but prevented the subsequent oligomerisation of Bax. Furthermore, CMV-encoded m38.5 and vMIA appear to block Bax downstream of translocation to mitochondria, when Bax has already undergone structural changes [125].

These data suggest that no universal mechanism exists that enables virus-encoded Bcl-2 to subvert premature host cell apoptosis, and that the precise mechanism reflects the unique circumstances under which viral infection takes place. In particular, the mechanism of action may be heavily influenced by the initial site of contact and tissue type. A pertinent example is CMV-encoded m38.5, with an m38.5 deletion virus showing no overt signs of impaired replication in visceral organs, whereas in salivary glands a 10–100 fold difference was observed [128]. This suggests that particular tissues may be more prone to infection; however, this aspect and the observation that expression patterns of pro-apoptotic Bcl-2 proteins vary amongst tissues has not been adequately addressed for the vast majority of viruses. Interestingly, viruses can manipulate the host cell apoptosis program on many levels, with evidence emerging that viruses can manipulate the caspase cascade [174] and the endogenous levels of the host Bcl-2 family members [175]. These findings add an additional layer of complexity to the quest of identifying the precise molecular mechanism of action of vBcl-2 proteins, and ultimately suggest that more sophisticated approaches may be required to answer these questions.

## Figures and Tables

**Table 1:** Pro-survival Bcl-2 proteins encoded by viruses.

Virus-Encoded Pro-Survival Bcl-2	Reference
γ-herpesviruses 68 M11	[45]
Adenovirus E1B19K	[46]
Epstein-Barr virus BHRF1	[34,47]
Epstein-Barr virus BALF1	[34,47]
Kaposi’s sarcoma-associated herpesvirus Ks-Bcl-2	[48,49]
Turkey herpesvirus vnr-13	[50]
African swine fever virus A179L	[36,51]
Grouper iridovirus GIV66	[52,53]
Myxoma virus M11L	[35,54]
Vaccinia virus F1L	[55,56,57]
Variola virus F1L	[58]
Ectromelia virus EMV025	[59]
Sheeppox virus SPPV14	[60]
Deerpox virus DPV022	[61,62]
Fowlpox virus FPV029	[63,64]
Canarypox CNP058	[65]
Lumpy skin disease virus LD17	[60]
Orfvirus ORFV125	[66]

**Table 2:** Affinities (in nM) of different pro-survival Bcl-2 proteins for peptides spanning the BH3 motif of endogenous pro-apoptotic Bcl-2 family members or Beclin-1 (measurements taken from: [34,35,36,57,58,60,62,64,110,137,138,139,140,141,142]).

	***Poxviral Bcl-2***					
**Pro-death**	SPPV14	M11L	MVA_F1L	VAR_F1L	DPV022	FPV039
**Bad**	>2000	>1000	NB	NB	NB	653
**Bid**	341	100	NB	3200	NB	2
**Bik**	>2000	>1000	NB	NB	NB	30
**Bim**	26	5	250	NB	340	10
**Bmf**	67	100	NB	NB	NB	16
**Hrk**	63	>1000	NB	NB	NB	24
**Noxa**	>2000	>1000	NB	NB	NB	28
**Puma**	65	>1000	NB	NB	NB	24
**Bak**	46	50	4300	2640	6930	76
**Bax**	32	75	1850	960	4040	76
**Beclin-1**	n/a	n/a	n/a	n/a	NB	n/a
	***Asfarviral Bcl-2***	***Herpesviral Bcl-2***				
	A179L	BHRF1	Ks-Bcl-2	M11	N1L	
**Bad**	258	>2000	>1000	NB	>1000	
**Bid**	26	109	112	232	152	
**Bik**	190	>2000	>1000	NB	n/a	
**Bim**	6	18	29	131	72	
**Bmf**	254	>2000	>1000	300	n/a	
**Hrk**	1487	>1000	>1000	719	n/a	
**Noxa**	1575	>2000	>1000	132	n/a	
**Puma**	31	70	69	370	n/a	
**Bak**	29	150	<50	76.3	71	
**Bax**	26	1,400	980	690	n/a	
**Beclin-1**		n/a	n/a	40.2	n/a	
	***Human Bcl-2***					***Sponge Bcl-2***
	Bcl-2	Bcl-w	Bcl-x_L_	Mcl-1	A1	BHP2
**Bad**	16	30	5.3	>100,000	15,000	NB
**Bid**	6800	40	82	2100	1	NB
**Bik**	850	12	43	1700	58	NB
**Bim**	2.6	4.3	4.6	2.4	1	NB
**Bmf**	3	9.8	9.7	1100	180	NB
**Hrk**	320	49	3.7	370	46	3760
**Noxa**	>100,000	>100,000	>100,000	24	20	NB
**Puma**	3.3	5.1	6.3	5	1	NB
**Bak**	>1000	500	50	10	3	66
**Bax**	100	58	130	12	n/a	NB
**Beclin-1**	n/a	n/a	2300	n/a	n/a	n/a

MVA = modified vaccinia virus Ankara, VAR = variole virus, n/a = not available, NB = no binding.
